# Intracranial Infections in Neurological Surgery: The Changes of Circular RNA Expression and Their Possible Function Mechanism

**DOI:** 10.1155/2020/2536272

**Published:** 2020-05-02

**Authors:** Jiangtao Niu, Hongqi Xu, Bo Yang

**Affiliations:** ^1^Neurosurgery Department, First Affiliated Hospital of Zhengzhou University, Henan Province, China; ^2^Neurosurgery Department, Anyang People's Hospital, Henan Province, China

## Abstract

**Methods:**

circRNA expression was analysed in six cerebrospinal fluid (CSF) samples from three patients of the infectious and noninfectious phases using an Arraystar Human circRNA Array. Differentially altered circRNAs were validated by quantitative real-time polymerase chain reaction (qRT-PCR) in the 66 CSF samples of 33 patients of the infectious and noninfectious phases. *t*-test was used for statistical analysis. A bioinformatics analysis was employed to investigate the function mechanism of the circRNAs.

**Results:**

Firstly, 142 circRNAs were found significantly different in 6 CSF samples of the infection and noninfection phases of 3 patients. Fourteen circRNAs with the top largest fold changes were chosen from the 142 circRNAs for PCR validation in the same 6 CSF samples of 3 patients. Three circRNAs were selected to be validated in 60 CSF samples of 30 patients using the PCR test. In infection CSF, an upregulated hsa_circRNA_402632 and downregulated hsa_circRNA_008636 and hsa_circRNA_405481 were confirmed by PCR test. A bioinformatics analysis was used to investigate the function mechanism of the 3 circRNAs. hsa_circRNA_402632 is enriched in the insulin resistance pathway, the FoxO and AMPK signaling pathways are the most important pathways for hsa_circRNA_008636 gene expression, and hsa_circRNA_405481 is enriched in the endometrial cancer signaling pathway, Fc epsilon RI signaling pathway, and TGF-beta signaling pathway.

**Conclusions:**

hsa_circRNA_402632, hsa_circRNA_008636, and hsa_circRNA_405481 may be potential diagnostic markers for central nervous system infection after neurological surgery.

## 1. Introduction

Postoperative central nervous system infection (PCNSI) is a common and serious complication [1–5]. The incidence of PCNSI is 7.4% [6]. PCNSI usually leads to longer hospital stay, multiple surgeries, and large-scale use of antibiotics. PCNSI often warrants the use of high-grade antibiotics, eventually leading to higher medical costs and complex and critical medical care [7–9]. An early diagnosis and the initiation for appropriate treatment can effectively improve the prognosis of patients with PCNSI. The main pathogen causing PCNSI is gram-positive cocci and gram-negative bacilli. A CSF bacterial culture is a necessary diagnostic method, which usually takes at least three days and has low positive results. The cell counting examination of CSF is often affected by the blood in the CSF. Therefore, neither a bacterial culture nor cytological detection of CSF can meet the needs of an early diagnosis of PCNSI. circRNA is an endogenous noncoding RNA with a covalently closed cyclic structure which enables circRNA to resist exonucleases. circRNAs often have high stability and have been widely studied as diagnostic markers in many diseases, including neurodegenerative disorders, diabetes, and various types of cancers [10]. Fan et al. investigated the profiles of circRNAs in tilapia meningoencephalitis and identified circRNAs as new biomarkers for meningoencephalitis and neurodegenerative disorders [11]. In this study, circRNAs as a marker for early diagnosis of PCNSI were investigated by collecting 66 CSF samples from 33 patients in infectious and noninfectious phases.

## 2. Methods

**Figure 1:**
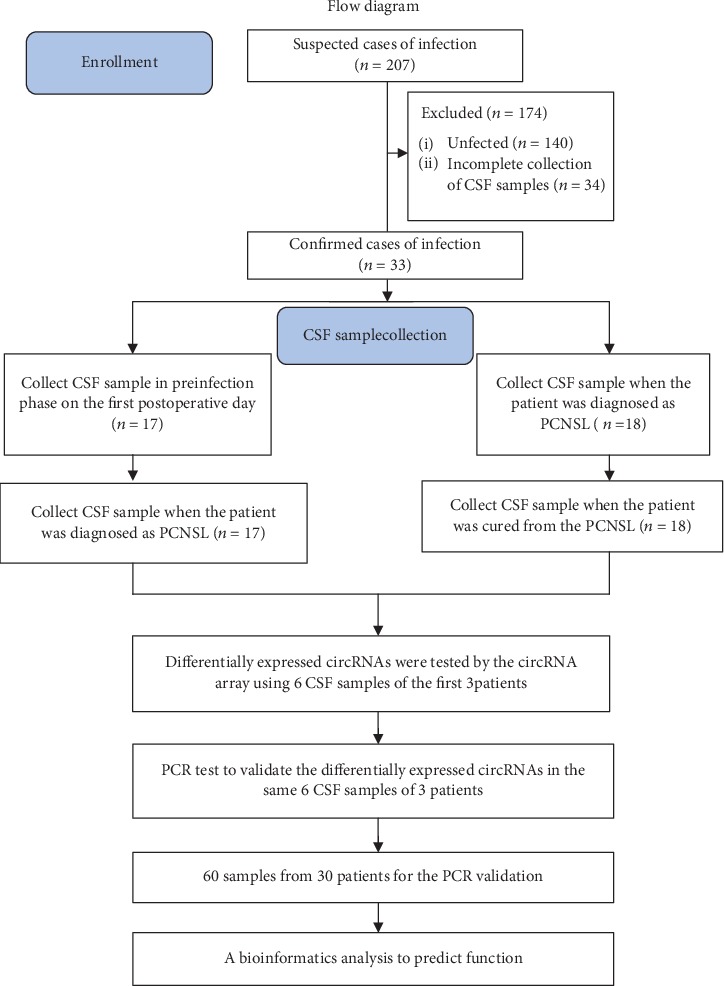
The flow diagram of this study.

Self-control samples were used for analysis from 33 selected patients at infected and noninfected phases from our center between February 2018 and November 2019. First, six CSF samples of three patients in infectious and noninfectious phases were obtained, and differentially expressed circRNAs were detected in the infection and noninfection groups by the Arraystar Human circRNA Array. Then, a qRT-PCR test was performed to validate the differentially expressed circRNAs with the same six CSF samples of the three patients used in the Arraystar Human circRNA Array. Following this, 60 CSF samples from 30 patients were prepared for the qRT-PCR test to validate the candidate circRNAs. The candidate circRNAs underwent a bioinformatics analysis to predict their molecular function and gene expression (Figure 1).

### 2.1. Ethics Statement

The study protocol was approved by the Ethics Committee of Anyang People's Hospital based on the ethical standards of the 1964 Declaration of Helsinki and its later amendments. The patients or their families were informed about the study, and their consent to participate in it was obtained.

### 2.2. Inclusion Criteria

The patients who enrolled in this study had central nervous system infection after neurological surgery therapy. The PCNSI was diagnosed according to the diagnostic criteria of the Centers for Disease Control and Prevention (CDC) [12]. Patients with central nervous system tumors were excluded from the study.

### 2.3. Clinical Characteristics of the Enrolled Patients

The study included 33 patients, 9 males (27.3%) and 24 females (72.7%). The average age was 58.88 ± 12.12 years (26 to 85 years). The initial diagnoses were cerebral hemorrhage in 20 patients (60.6%), subarachnoid hemorrhage (SAH) caused by intracranial aneurysm or AVM in 7 patients (21.3%), trigeminal neuralgia or facial spasm in 3 patients (9.1%), brain trauma in 1 case (3.0%), hydrocephalus in 1 case (3.0%), and cerebellar tonsillar hernia malformation with syringomyelia in 1 case (3.0%) (Table 1).

The neurological surgery therapy was ventricular drainage in 10 patients (30.3%), craniotomy combined with ventricular drainage in nine cases (27.2%), craniotomy in five cases (15.2%), ventricular drainage combined with lumbar drainage in three cases (9.1%), craniotomy combined with decompressive craniectomy in two cases (6.1%), lumbar drainage in two cases (6.1%), decompressive craniectomy in one case (3.0%), and a ventriculoperitoneal shunt in one case (3.0%).

Among them, 26 cases (78.8%) underwent an emergency operation, and seven cases (21.2%) underwent selective operation.

### 2.4. Sample Collection and Preparation

Sixty-six CSF samples of thirty-three patients in infectious and noninfectious phases were obtained. Among these, 17 noninfected CSF samples were obtained on the first day after neurological surgery when the patients were in uninfected phases; the infected CSF samples from the same 17 patients were obtained at the time of diagnosis of infection. In the other 18 patients, the infected CSF samples were obtained first at the time of diagnosis of infection, and the noninfected CSF samples from the same 18 patients were obtained after the PCNSL was cured.

Sixty-six CSF samples from thirty-three patients were collected, centrifuged at 3000 rpm for three minutes and frozen at -80°C in the refrigerator. Each sample was 5 ml in size. Among these, 6 CSF samples from 3 patients were used for circRNA profiling using the Arraystar Human circRNA Array. The criteria for selecting patients for circRNA array were random; any patient enrolled in our study might be selected. For the convenience of the experiment, we chose the first three patients enrolled in our study for circRNA array. First, the total RNA extracted and purified was quantified using the NanoDrop ND-1000.

### 2.5. circRNA Microarray Detection

Microarray hybridization was performed based on the Arraystar's standard protocols. The RNAs extracted were digested with RNase R (Epicentre, Inc.) to remove linear RNAs and enrich circular RNAs. Then, the enriched circular RNAs were amplified and transcribed into fluorescent circRNAs utilizing a random priming method (Arraystar Super RNA Labeling Kit; Arraystar). The labeled circRNAs were hybridized onto the Arraystar Human circRNA Array V2 (8x15K, Arraystar). After having washed the slides, the arrays were scanned by the Agilent Scanner G2505C. Agilent Feature Extraction software (version 11.0.1.1) was used to analyze the acquired array images. Quantile normalization and subsequent data processing were performed using the R software limma package. Differentially expressed circRNAs with statistical significance between the noninfection and infection groups were identified through volcano plot filtering. Differentially expressed circRNAs between the noninfection and infection groups were identified through fold-change filtering. Hierarchical clustering was performed to show the distinguishable circRNA expression pattern among samples.

### 2.6. Polymerase Chain Reaction Validation

Primers were designed and synthetized from the six samples which were used for circRNA profiling, and these were also used for the PCR experiment to validate the preliminary and candidate circRNAs. Sixty samples from thirty patients were prepared for PCR of the expanded sample size to validate the candidate circRNAs.

### 2.7. Bioinformatics Analysis

CircInteractome through the NIH web was used to analyze RNA-binding protein sites that matched with circRNAs and analyze circRNAs for predictions through a microRNA (miRNA) sponge. According to the endogenous RNA (ceRNA) mechanism, the TargetScan, miRTarBase, and miRDB databases were used to analyze the target proteins for miRNA, and R software was used to analyze all the target proteins that bind to miRNA. Gene Ontology (GO) and Kyoto Encyclopedia of Genes and Genomes (KEEG) pathway analyses were used to identify the ceRNA target proteins' function and signal pathways.

### 2.8. Statistical Analysis

When comparing differences of circRNA expression profiling between the infected and noninfected groups, the fold change between the groups for each circRNA was computed. The statistical significance of the difference was analyzed by *t*-test. circRNAs having fold changes ≥ 1.5 and *p* values ≤ 0.05 are selected as the significantly differentially expressed. We filtered the analysis outputs and ranked the differentially expressed circRNAs according to fold change and *p* value, using Microsoft Excel's Data/Sort & Filter functionalities.

## 3. Results

### 3.1. Screening of Differentially Expressed circRNAs

**Figure 2:**
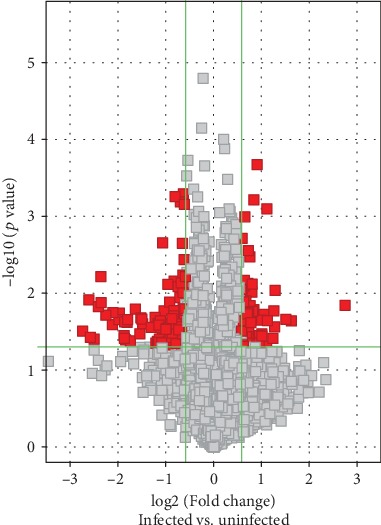
Volcano plot: volcano plots were used to identify differentially expressed circRNAs in infected CSF vs. noninfected CSF. The *x*-axis represents fold-change values (log2-scaled), while the *y*-axis represents *p* values (−log10-scaled). The green vertical lines correspond to 1.5x upregulation and downregulation, while the green horizontal line corresponds to a *p* value of 0.05. On this basis, the red rectangles represent the differentially expressed circRNAs with statistical significance.

**Figure 3:**
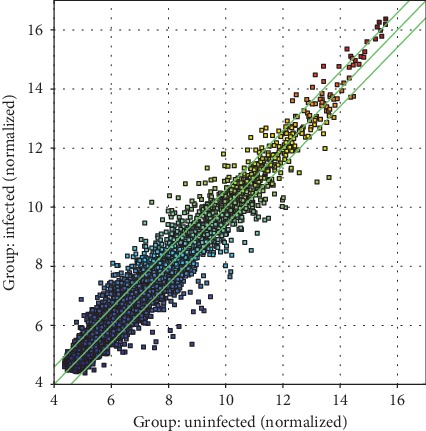
Scatter plot: scatter plots used to identify differentially expressed circRNAs in infected CSF vs. noninfected CSF. The *x*-axis and *y*-axis represent the mean normalized circRNA signal values for each comparator group (log2-scaled). The green fold-change lines represent 1.5x fold changes, so the circRNAs lying above and below these green lines displayed greater than a 1.5-fold upregulation or downregulation, respectively.

**Figure 4:**
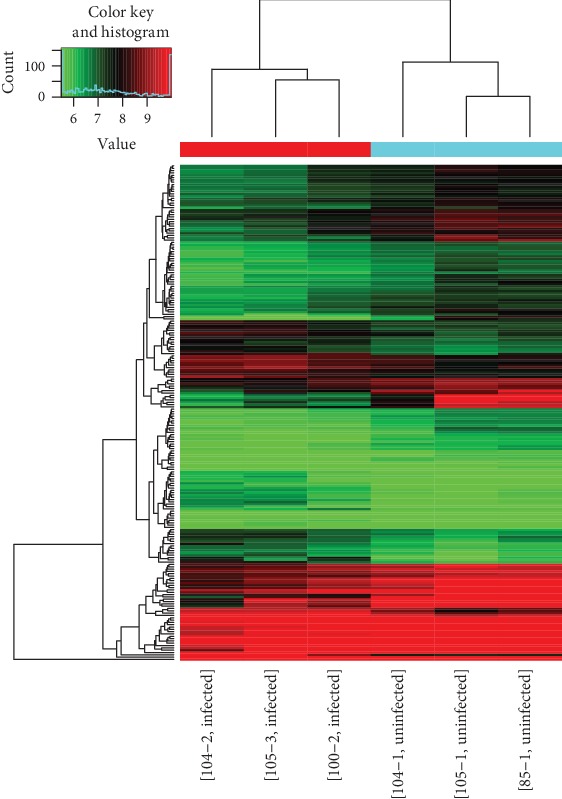
Hierarchical clustering used to show the distinguishable circRNA expression pattern among samples. Hierarchical clustering showed that the infection group and noninfection group have clustering.

The circRNA array detected a large number of differentially expressed circRNAs, of which 6141 were upregulated and 6446 were downregulated. circRNAs having fold changes ≥ 1.5 and *p* values ≤ 0.05 were selected as the significantly differentially expressed circRNAs. A volcano plot was used to visualize the differential expression of circRNAs between noninfection CSF and infection CSF groups (Figure 2). The scatter plot was employed to assess the circRNA expression variation between noninfection CSF and infection CSF groups (Figure 3). Hierarchical clustering was performed to hypothesize the relationships between samples based on differentially expressed circRNAs, and the result of hierarchical clustering showed a distinguishable circRNA expression profiling among samples (Figure 4). The results when compared between the infected and noninfected groups showed that the number of upregulated circRNA with a significant difference was 72 and the number of downregulated circRNA was 142.

### 3.2. PCR Validation of Candidate circRNAs

Based on the results of the previous circRNA array, 14 circRNAs with the largest significant differences and the top largest fold changes were selected for PCR validation. The upregulated circRNAs were hsa_circRNA_402768, hsa_circRNA_402632, hsa_circRNA_061260, hsa_circRNA_048410, hsa_circRNA_048148, and hsa_circRNA_402482, and the downregulated circRNAs were hsa_circRNA_055243, hsa_circRNA_101900, hsa_circRNA_405481, hsa_circRNA_007059, hsa_circRNA_101366, hsa_circRNA_102213, hsa_circRNA_008636, and hsa_circRNA_009012. A qRT-PCR test was performed using the 6 samples from the 3 patients used in the Arraystar Human circRNA Array to validate the circRNAs (Table 2).

The PCR-validated results of the 6 samples showed that of the 6 upregulated circRNAs in the previous circRNA array, the changes of two circRNAs did not correspond with the circRNA array result. The four remaining circRNAs corresponded with the circRNA array result, but the *p* value was above 0.05. The discrepancy in the *p* value could be attributed to the fact that the number of validation samples was relatively small and the intragroup difference was large, which led to a large *p* value. The subsequent selection of candidate circRNAs and the validation of an expanded sample size might lead to the *p* value meeting the standard of less than 0.05. Considering that the *p* value was as close as possible to 0.05, the alternative upregulated circRNA was hsa_circRNA_402632. The PCR-validated results of the 6 samples showed that among the 8 downregulated circRNAs, the *p* value of one circRNA did not correspond with the circRNA array result and the remaining seven circRNAs corresponded with the circRNA array result. Considering that the *p* value is less than 0.05, the alternative downregulated circRNAs were hsa_circRNA_008636 and hsa_circRNA_405481. Subsequently, we expanded the sample size for validation; 30 patients were included, and each patient had two CSF samples taken, infected and noninfected. The PCR results of the expanded sample size showed that the former three circRNAs corresponded with the circRNA array result. Therefore, hsa_circRNA_402632, hsa_circRNA_008636, and hsa_circRNA_405481 can be considered as diagnostic markers of PCNSI (Table 3).

### 3.3. Function Mechanism of Candidate circRNAs

For hsa_circRNA_008636, the CircInteractome analysis showed that there were 12 RNA-binding proteins and 32 sponge miRNAs that could bind to themselves, and 6 other sponge miRNAs were recommended by the array company. The total number of genes which could be targeted with miRNAs was 526. All the proteins were analyzed by KEEG pathway and GO analyses. According to the KEEG pathway analysis, there were four pathways with a similarity score above 0.5: the FoxO signaling pathway, the AMPK signaling pathway, the pancreatic cancer pathway, and the colorectal cancer pathway. The first two pathways, FoxO and AMPK, were important in oxidative stress and inflammatory response.

As there was no registration for hsa_circRNA_402632 in the circBase, the CircInteractome analysis could not be carried out. According to 6 sponge miRNAs recommended by the array company, the TargetScan, miRTarBase, and miRDB databases were used to analyze the target proteins, and 114 genes could be targeted with the miRNAs. Ultimately, the KEEG pathway and GO analyses were performed, and only the insulin resistance pathway could be enriched by four genes. As for hsa_circRNA_405481, a new circRNA, 29 genes could be targeted with sponge miRNAs. The KEEG pathway analysis showed that the endometrial cancer signaling pathway, Fc epsilon RI signaling pathway, and TGF-beta signaling pathway could be enriched.

## 4. Discussion

Central nervous system infections have always been a big problem for doctors and patients following neurological surgery, a result of which the financial burden on patients also increases [13]. In severe cases, which may lead to death or severe disability, the burden of this disease increases further [14]. For a long time, there has been a lack of good early diagnostic indicators of intracranial infection after neurological surgery [15]. CSF is the main test sample for intracranial infection [16]. A bacterial culture of CSF usually takes three days, and it has a low positive rate. The cytological detection of CSF is often affected by the blood in the CSF. As such, neither of these methods can be relied upon to meet the needs of an early diagnosis. An early diagnosis is helpful to the prognosis of patients, and therefore, it is important to find early diagnostic markers of intracranial infection. circRNA is an exceptionally stable form of RNA that cannot be degraded easily, and it has great potential as a diagnostic marker due to its conservation, abundance, cell type-specific expression, tissue-specific expression, and it roles in disease progression [17–19]. Akhter has provided evidence that ciRS-7 has the potential as a biomarker for neurodegenerative disorders [20]. Furthermore, hsa_circRNA_103636 has been proven as a potential novel diagnostic and therapeutic biomarker in major depressive disorder [21], and Zhao et al. have proven that MMD-specific circRNAs are potential biomarkers and therapeutic targets [22]. Above all, circRNA exists widely in the central nervous system [23, 24] of patients and can be used as a good indicator of intracranial infection [25].

Fan et al. investigated the profiles of circRNAs in tilapia meningoencephalitis and identified circRNAs as new biomarkers for meningoencephalitis, which indicates that circRNA has the potential to be a biomarker of PCNSL in humans. However, it is difficult to obtain brain tissue from patients with PCNSL; therefore, CSF sample biomarkers for PCNSL for routine testing would be more suitable. In our study, the circRNA array detected a large number of differentially expressed circRNAs in infected CSF, of which, 6141 were upregulated and 6446 were downregulated. Top significant differences, top largest fold changes, and the least *p* value served as the selection criteria and main reference index. Six upregulated and eight downregulated circRNAs were selected for PCR validation of the six CSF samples used in the circRNA array. Eventually, one upregulated and two downregulated circRNAs were selected for PCR validation of the 60 CSF samples from 30 patients. Our results suggest that the CSF sample can be used as a sample for the detection of circRNA. In addition, there are significant differences in the expression of three particular circRNAs in the infected CSF when compared to the expression of circRNAs in the noninfected CSF. These three particular circRNAs have the potential to serve as biomarkers of PCNSI.

Among 33 noninfected CSF samples of 33 patients, 17 CSF samples were preinfected which were obtained on the first day after neurological surgery. In the other 18 patients, the CSF samples were in the postinfected phase, which were obtained after the PCNSL was cured. Therefore, the two groups of noninfected CSF samples belonged to different postoperative phases: one was the preinfection phase and the other was the postinfection phase. Finally, three circRNAs were screened by the circRNA array and validated by PCR; there were significant differences in the expression of three circRNAs between infected CSF and noninfected CSF. In particular, the noninfected CSF samples belonged to different postoperative phases; as a result of that, there was no correlation between the expression changes of circRNAs and postsurgical reaction; similarly, there was no correlation between the expression changes of circRNAs and the blood in CSF.

The function of the three circRNAs was not clear; therefore, a bioinformatics analysis was conducted, of which, the RNA-binding protein analysis and ceRNA analysis were the most important ways to investigate circRNA. The target protein may predict circRNA's function. For hsa_circRNA_008636, according to the KEEG pathway analysis, FoxO and AMPK signaling pathways are the most important pathways, which predict that hsa_circRNA_008636 may be related to oxidative stress and inflammatory response. hsa_circRNA_402632 has the potential to enrich the insulin resistance pathway and hsa_circRNA_405481 affects the endometrial cancer signaling pathway, Fc epsilon RI signaling pathway, and TGF-beta signaling pathway, both of which may be related to inflammatory response. Therefore, the three circRNAs have a significant underlying application prospect in the diagnosis of intracranial infection.

Our study confirmed that hsa_circRNA_402632, hsa_circRNA_405481, and hsa_circRNA_008636 have significant differences in their expression when compared between infected and noninfected CSF samples. The three circRNAs have not been proven to have any function in vivo prior to our study being conducted. In our study, these three circRNAs had been detected by circRNA microarray and validated with CSF samples by a PCR test twice, and therefore, they can be used as reliable diagnostic markers for PCNSI. These results can be applied in everyday practice as the differences in the expression of circRNA in CSF can enable a diagnosis of PCNSI. A large enough fold change in the expression difference is indicative of the presence of infection. It is important to note that at the time of sample collection, all the patients were using different kinds of antibiotics either to prevent or to treat pulmonary infections and other infections. As such, the diagnostic results of circRNA as a diagnostic marker are not affected in the presence of antibiotics.

A few limitations of the study meant that we could not produce the circRNA kit and have also not carried out clinical trials on a large number of patients. However, this present study is the basis for further research.

With the development of molecular biology and bioinformatics knowledge, various laboratory-related diagnoses of intracranial infection are also being developed. circRNA has a highly conserved sequence and plays a role in gene regulation as RNA-binding proteins (RBPs), altering RBP activity, miRNA sponge activity, and rolling circle translation. It also demonstrates conservation, abundance, cell type-specific expression, and tissue-specific expression and participates in the development of various diseases. Therefore, it will gradually be used as a new clinical diagnostic marker or a treatment target for many diseases such as intracranial infection.

## 5. Conclusion

In this study, the circRNA array detected a large number of differentially expressed circRNAs between noninfected and infected CSF (6141 upregulated and 6446 downregulated). A qRT-PCR test was used to validate the differentially expressed circRNAs. Finally, three circRNAs were identified, including hsa_circRNA_402632, hsa_circRNA_405481, and hsa_circRNA_008636. A bioinformatics analysis was conducted to explore the potential functions of these circRNAs, which revealed the following results: hsa_circRNA_008636 can enrich the FoxO signaling pathway and the AMPK signaling pathway and hsa_circRNA_402632 can enrich the insulin resistance pathway, while hsa_circRNA_405481 can enrich the endometrial cancer signaling pathway, Fc epsilon RI signaling pathway, and TGF-beta signaling pathway, all of which may be related to oxidative stress and inflammatory response. Therefore, they have a strong underlying application prospect as biomarkers in the diagnosis of intracranial infection.

## Figures and Tables

**Table 1:** The general information of the patients enrolled in the study.

The primary diseases	Number of infected	Proportion	Number of cured
Cerebral hemorrhage	20	60.6%	16
SAH caused by intracranial aneurysm or AVM	7	21.3%	7
Trigeminal neuralgia or facial spasm	3	9.1%	3
Brain injury	1	3.0%	1
Hydrocephalus	1	3.0%	1
Arnold-Chiari malformation	1	3.0%	1

^1^SAH: subarachnoid hemorrhage; AVM: ^2^Arteriovenous malformation.

**Table 2:** The result of PCR of 6 CSF samples.

	Noninfected group			Infected group			Fc^1^	*p* value
hsa_circRNA_007059/*β*-actin	5.49*E*-02	4.29*E*-02	7.24*E*-02	1.06*E*-02	9.36*E*-03	1.17*E*-02	0.19	0.00581
hsa_circRNA_009012/*β*-actin	1.30*E*-02	1.80*E*-02	1.06*E*-02	5.77*E*-03	9.04*E*-03	2.32*E*-02	0.91	0.84592
hsa_circRNA_055243/*β*-actin	1.32*E*-01	7.34*E*-02	8.09*E*-02	1.07*E*-02	3.65*E*-02	4.96*E*-02	0.34	0.04358
hsa_circRNA_101900/*β*-actin	3.40*E*-02	2.30*E*-02	2.11*E*-02	5.79*E*-03	1.26*E*-02	1.54*E*-02	0.43	0.03993
hsa_circRNA_102213/*β*-actin	2.10*E*-01	1.79*E*-01	1.30*E*-01	4.56*E*-02	8.46*E*-02	1.20*E*-01	0.48	0.0481
hsa_circRNA_402632/*β*-actin	2.62*E*-01	1.04*E*-01	3.06*E*-01	3.40*E*-01	9.24*E*-01	3.69*E*-01	2.43	0.18397
hsa_circRNA_402768/*β*-actin	2.27*E*-02	2.32*E*-02	1.66*E*-02	6.41*E*-03	1.30*E*-01	4.42*E*-02	2.89	0.34296
hsa_circRNA_405481/*β*-actin	1.37*E*-02	1.51*E*-02	9.97*E*-03	1.93*E*-03	7.73*E*-03	5.70*E*-03	0.4	0.02691
hsa_circRNA_008636/*β*-actin	6.46*E*-03	1.05*E*-02	7.25*E*-03	1.14*E*-03	1.03*E*-04	2.22*E*-03	0.14	0.00724
hsa_circRNA_048148/*β*-actin	1.92*E*-02	2.10*E*-02	1.68*E*-02	7.33*E*-05	1.73*E*-04	2.26*E*-04	0.01	0.00011
hsa_circRNA_402482/*β*-actin	1.22*E*-02	1.62*E*-02	1.67*E*-04	1.48*E*-02	1.75*E*-02	1.59*E*-02	1.69	0.25121
hsa_circRNA_101366/*β*-actin	8.24*E*-02	6.36*E*-02	4.22*E*-02	7.01*E*-03	1.56*E*-03	2.04*E*-03	0.06	0.00729
hsa_circRNA_061260/*β*-actin	2.27*E*-02	4.61*E*-02	3.36*E*-02	2.85*E*-02	2.44*E*-02	3.30*E*-02	0.84	0.48882
hsa_circRNA_048410/*β*-actin	3.69*E*-02	4.92*E*-02	4.85*E*-02	5.36*E*-02	9.92*E*-02	1.19*E*-01	2.02	0.08127

^1^Fold change: infected group/noninfected group.

**Table 3:** The result of PCR of the expanded sample size.

circRNAs	Fc (infected group/noninfected group)	*p* value
hsa_circRNA_402632/*β*-actin	2.065053374	0.000816666
hsa_circRNA_008636/*β*-actin	0.415755565	0.000117669
hsa_circRNA_405481/*β*-actin	0.451595624	0.000151069

## Data Availability

The data used to support the findings of this study are available from the corresponding author upon request.

## References

[1] Yadegarynia D., Gachkar L., Fatemi A. (2014). Changing pattern of infectious agents in postneurosurgical meningitis. *Caspian Journal of Internal Medicine*.

[2] McClelland S., Hall W. A. (2007). Postoperative central nervous system infection: incidence and associated factors in 2111 neurosurgical procedures. *Clinical Infectious Diseases*.

[3] Sneh-Arbib O., Shiferstein A., Dagan N. (2013). Surgical site infections following craniotomy focusing on possible post-operative acquisition of infection: prospective cohort study. *European Journal of Clinical Microbiology & Infectious Diseases*.

[4] Neuberger A., Shofty B., Bishop B. (2016). Risk factors associated with death or neurological deterioration among patients with Gram-negative postneurosurgical meningitis. *Clinical microbiology and Infection*.

[5] Dashti S. R., Baharvahdat H., Spetzler R. F. (2008). Operative intracranial infection following craniotomy. *Neurosurgical focus*.

[6] Zhan R., Zhu Y., Shen Y. (2014). Post-operative central nervous system infections after cranial surgery in China: incidence, causative agents, and risk factors in 1,470 patients. *European Journal of Clinical Microbiology & Infectious Diseases*.

[7] Ma Y.-F., Wen L., Zhu Y. (2017). Prospective study evaluating post-operative central nervous system infections following cranial surgery. *British journal of neurosurgery*.

[8] Kourbeti I. S., Vakis A. F., Ziakas P. (2015). Infections in patients undergoing craniotomy: risk factors associated with post-craniotomy meningitis. *Journal of neurosurgery*.

[9] Yao J., Liu D. (2019). Logistic regression analysis of risk factors for intracranial infection after multiple traumatic craniotomy and preventive measures. *The Journal of craniofacial surgery*.

[10] Xu S., Zhou L., Ponnusamy M. (2018). A comprehensive review of circRNA: from purification and identification to disease marker potential. *PeerJ*.

[11] Fan B., Chen F., Li Y. (2019). A comprehensive profile of the tilapia (Oreochromis niloticus) circular RNA and circRNA-miRNA network in the pathogenesis of meningoencephalitis of teleosts. *Molecular omics*.

[12] Horan T. C., Gaynes R. P., Martone W. J., Jarvis W. R., Emori T. G. (1992). CDC definitions of nosocomial surgical site infections, 1992: a modification of CDC definitions of surgical wound infections. *Infection control and hospital epidemiology*.

[13] Kourbeti I. S., Jacobs A. V., Koslow M., Karabetsos D., Holzman R. S. (2007). Risk factors associated with postcraniotomy meningitis. *Neurosurgery*.

[14] Erdem I., Hakan T., Ceran N. (2008). Clinical features, laboratory data, management and the risk factors that affect the mortality in patients with postoperative meningitis. *Neurology India*.

[15] Yu Y., Li H. J. (2017). Diagnostic and prognostic value of procalcitonin for early intracranial infection after craniotomy. *Brazilian journal of medical and biological research = Revista brasileira de pesquisas medicas e biologicas*.

[16] Ruan L., Wu D., Li X. (2017). Analysis of microbial community composition and diversity in postoperative intracranial infection using high-throughput sequencing. *Molecular medicine reports*.

[17] Danan M., Schwartz S., Edelheit S., Sorek R. (2012). Transcriptome-wide discovery of circular RNAs in Archaea. *Nucleic acids research*.

[18] Suzuki H., Tsukahara T. (2014). A view of pre-mRNA splicing from RNase R resistant RNAs. *International journal of molecular sciences*.

[19] Bahn J. H., Zhang Q., Li F. (2015). The landscape of microRNA, Piwi-interacting RNA, and circular RNA in human saliva. *Clinical chemistry*.

[20] Akhter R. (2018). Circular RNA and Alzheimer's disease. *Advances in experimental medicine and biology*.

[21] Cui X., Niu W., Kong L. (2016). hsa_circRNA_103636: potential novel diagnostic and therapeutic biomarker in major depressive disorder. *Biomarkers in medicine*.

[22] Zhao M., Gao F., Zhang D. (2017). Altered expression of circular RNAs in Moyamoya disease. *Journal of the neurological sciences*.

[23] Rybak-Wolf A., Stottmeister C., Glažar P. (2015). Circular RNAs in the mammalian brain are highly abundant, conserved, and dynamically expressed. *Molecular Cell*.

[24] You X., Vlatkovic I., Babic A. (2015). Neural circular RNAs are derived from synaptic genes and regulated by development and plasticity. *Nature neuroscience*.

[25] Meng S., Zhou H., Feng Z. (2017). circRNA: functions and properties of a novel potential biomarker for cancer. *Molecular Cancer*.

